# ZnO based photocatalysts for pesticides degradation

**DOI:** 10.1039/d5ra08016a

**Published:** 2025-12-15

**Authors:** Haji Muhammad, Maliha Hanif, Mustafa Tuzen, Asma Siddiqui, Nazish Kousar, Afzal Shah, Mohammad Reza Afshar Mogaddam

**Affiliations:** a Chemistry Department, Faculty of Science and Arts, Tokat Gaziosmanpasa University 60250 Tokat Turkey mustafa.tuzen@gop.edu.tr; b Department of Chemistry, University of Karachi Karachi 75270 Pakistan; c Department of Chemistry, Federal Urdu University of Arts, Sciences and Technology Karachi 75300 Pakistan; d Department of Biotechnology, Federal Urdu University of Arts, Sciences and Technology Karachi 75300 Pakistan; e Department of Chemistry, Quaid-i-Azam University Islamabad 45320 Pakistan afzals_qau@yahoo.com; f Food and Drug Safety Research Center, Pharmaceutical Sciences Institute, Tabriz University of Medical Sciences Tabriz Iran; g Research Center of New Material and Green Chemistry, Khazar University 41 Mehseti Street Baku AZ1096 Azerbaijan

## Abstract

The widespread application of pesticides in modern agriculture has significantly boosted crop production; however, their inherent toxicity, persistence, and resistance to conventional cleanup methods have led to serious environmental and public health concerns. Advanced oxidation processes (AOP), especially those utilizing visible light for photocatalysis, have recently emerged as promising eco-friendly alternatives for the degradation of pesticides. In particular, zinc oxide (ZnO) based nanophotocatalysts have garnered considerable attention due to their wide band gap (∼3.37 eV), strong oxidative capability, high electron mobility, low electron–hole recombination rates, and natural antibacterial properties, which enhance their photocatalytic activity under sunlight. This review provides a comprehensive overview of recent progress in ZnO-mediated photocatalytic degradation of pesticides, focusing on synthesis methods, structural modifications such as doping and defect engineering, and material hybridization aimed at improving photocatalytic efficiency. Furthermore, the study critically examines the influence of key factors, including catalyst concentration, surface morphology, and particle size, on degradation performance. This review aims to offer a thorough understanding of the versatility of ZnO as a tunable photocatalyst for mitigating pesticide contamination in wastewater by combining mechanistic insights with experimental observations. This integration not only highlights the potential of ZnO in this context but also establishes a foundation for creating scalable and eco-friendly remediation approaches.

## Abstract 2

The widespread application of pesticides in modern agriculture has significantly boosted crop production; however, their inherent toxicity has serious environmental and public health concerns.

## Graphical abstract

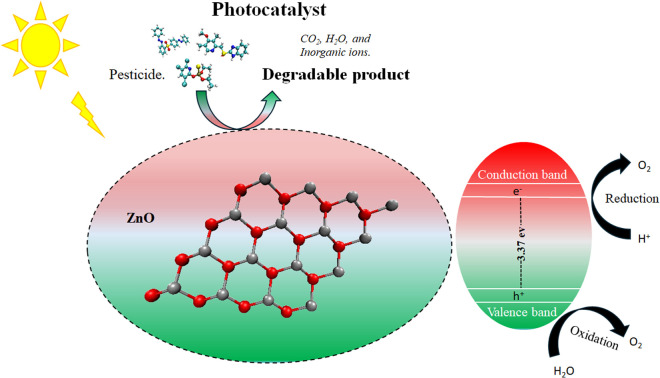

## 1. Introduction

Pesticides play a vital role in modern agriculture, serving as indispensable tools for farmers to manage and mitigate weeds and control pests in farming practices. They play a significant role in combating insect-borne diseases and are also believed to greatly enhance agricultural productivity by reducing crop losses, improving yield, and ensuring the quality of food, all while being a cost-effective solution for farmers.^1^ The application of pesticides on crops is estimated to be 3.5 million tons every year at a global scale. Despite the fact that approximately 1% of pesticides are effectively used to target and manage pests on the intended crops, the vast majority of these chemicals end up affecting non-target plants or dispersing into the environment. This unintended distribution leads to significant contamination of soil, water, and air, posing serious risks to ecosystems and public health.^2^ Consequently, the widespread use of pesticides disrupts food webs in both terrestrial and aquatic ecosystems, as the chemicals and their byproducts are often resistant to biodegradation, leading to persistent environmental contamination.^3^ Additionally, these agrochemicals contribute to a reduction in biodiversity, a decrease in pollinator populations, harm to native soil microorganisms, and the disturbance of nesting habitats.^4^

Moreover, pesticide exposure is linked to a variety of health problems, affecting individuals in environmental, community, and occupational settings. These health issues can manifest as both acute and chronic effects, including cancer, genetic mutations, neurotoxicity,^5^ and developmental disorders.^6^ It is estimated that annually, more than a million agricultural workers show signs of pesticide poisoning.^7^ As a result, the breakdown of pesticides and their residues is crucial. Many research efforts focus on techniques to minimize pesticide levels in the environment, particularly in soil and water, employing methods such as membrane filtration, surface adsorption, and biological degradation. However, these approaches may have limitations when faced with high levels of contamination.^8^

Nanoparticles have garnered significant attention for their potential in pesticide degradation, with ZnO nanoparticles being particularly notable due to their abundance, stability, high reactivity, large surface area, excellent photosensitivity, and cost-effectiveness.^9^ Furthermore, zinc oxide (ZnO) photocatalysts can be easily immobilized on various substrates, enhancing their applicability in diverse water treatment processes.^10^ These advantageous characteristics drive researchers to innovate and create advanced ZnO hybrid photocatalysts with improved photo-efficiency, aimed at effectively breaking down hazardous pollutants such as pesticides.

Therefore, in view of the aforesaid, this review employed a narrative literature approach, primarily sourcing scientific publications, review articles, and reports from online databases such as Google Scholar, ScienceDirect, Springer, PubMed, and Scopus. Key terms included “ZnO photocatalysis”, “pesticide degradation”, “nanoparticle remediation”, and “environmental impact of pesticides”. While studies published in peer-reviewed journals over the past 10 to 15 years were prioritized, the review did not impose strict inclusion or exclusion criteria. It aims to provide a comprehensive understanding of ZnO-mediated photocatalytic pesticide degradation by integrating both theoretical and experimental findings, alongside relevant earlier research that contributes valuable context or mechanistic insights. Only English-language publications specifically addressing ZnO-based photocatalytic pesticide breakdown were considered, while patents, conference abstracts, and non-peer-reviewed materials were excluded. Moreover, this manuscript was proofread and edited for clarity and language enhancement using ChatGPT; OpenAI. The authors assume full responsibility for all content and ensure the accuracy and integrity of the work.

## 2. Environmental fate and impact of pesticides

Pesticides are man-made chemical substances that include nematicides, herbicides, fungicides, acaricides, insecticides, and molluscicides. These compounds are employed to control, eliminate, reduce, or repel organisms that are detrimental to crops or cause damage. Their absence would result in a startling 78% loss in fruit productivity, a 54% decrease in vegetable production, and a 32% decrease in cereal production.^11^ Interestingly, pesticide use skyrocketed during World War II as a result of the increased need for food by a growing population. Initially, synthetic pesticides were primarily designed to eliminate mosquitoes, particularly those that carry malaria.^12^ But after the 1950s, global pesticide production has seen growth at an average annual rate of approximately 11%, escalating from 0.2 million tons (ref. 13) to over 5 million tons by the year 2000.^14^ Whereas annually, around 3 billion kilograms of pesticides are utilised globally.^15^

**Fig. 1:**
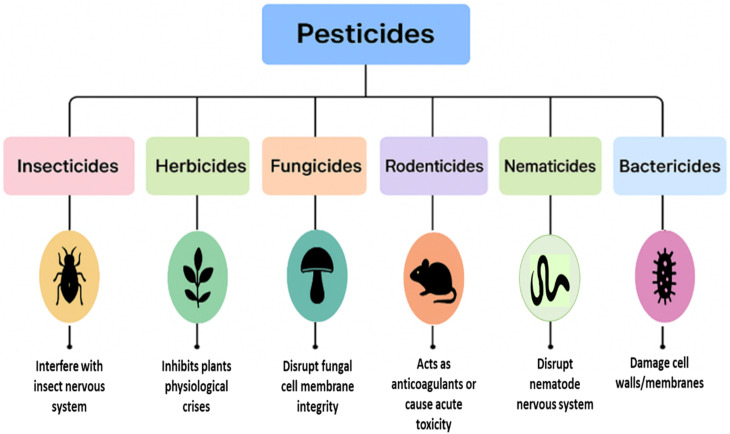
Classification of pesticides based on the targeted pest.

Likewise, synthetic pesticides have been used by humans since 1940; ever since, they vary in chemical, physical, and other characteristics from one category to another. Hence, it is important to classify them according to their properties and study them within their specific category. At present, pesticides can be broadly categorized in two ways: (a) based on the type of pest they target (Fig. 1) and (b) according to their chemical composition (Table 1).

**Table 1:** Classification of pesticides based on chemical groups and relevance to ZnO-mediated photocatalysis^a^

Chemical group	Examples	Structure type	Relevance to ZnO photocatalysis	Ref.
Organochlorines	DDT, aldrin, endosulfan, chlordane, heptachlor	Chlorinated aromatic hydrocarbons, high stability	Persistent organic pollutants require prolonged photocatalytic exposure to break C–Cl bonds	16
Organophosphates	Malathion, parathion, chlorpyrifos, diazinon	Phosphorothioates or phosphates, aliphatic or aromatic	Degrade relatively quickly; P–O and P–S bonds cleaved under UV/visible light	17
Carbamates	Carbaryl, aldicarb, carbofuran, methomyl	Carbamate esters of aromatic/aliphatic amines	ZnO photocatalysis breaks carbamate linkages, reducing toxicity	18
Pyrethroids	Permethrin, cypermethrin, deltamethrin	Cyclopropane carboxylate esters	Degrade into less toxic acids and alcohols under photocatalytic conditions	19
Neonicotinoids	Imidacloprid, thiamethoxam, acetamiprid	Chloronicotinyl or nitroguanidine derivatives	Water-soluble; susceptible to oxidative degradation in aqueous ZnO systems	20
Phenoxy herbicides	2,4-D, MCPA, mecoprop	Chlorinated phenoxyacetic acids	Aromatic rings and –COOH groups are readily attacked by OH˙	21
Triazines	Atrazine, simazine, propazine	Nitrogen-containing heterocyclic aromatic rings	Photocatalysis disrupts the triazine ring structure and dechlorinates	22
Bipyridyl herbicides	Paraquat, diquat	Bipyridinium salts	Strongly absorb visible light; photocatalysis reduces toxicity *via* demethylation and ring cleavage	23
Fumigants	Methyl bromide, aluminium phosphide	Simple halides or phosphides	Easily decomposed into non-toxic products under photocatalytic oxidation	24
Rodenticides	Warfarin, brodifacoum	Coumarin derivatives	Aromatic and lactone structures oxidised under UV/visible photocatalysis	25

a DDT = dichlorodiphenyltrichloroethane. 2,4-D = 2,4-dichlorophenoxyacetic acid. MCPA = 2-methyl-4-chlorophenoxyacetic acid.

However, the overuse of pesticides poses a significant risk to non-target organisms, as nearly 98% of all sprayed pesticides, whether directly or indirectly, impact them. Evidence suggests that soil and water quality deteriorate due to pesticide accumulation. Moreover, pesticide accumulation can lead to a decrease in soil respiration up to 35%. Existing literature indicates that nearly 90% of water sources in agricultural areas are polluted with pesticides.^26^

What is more, in the presence of various existing persistent pollutants (industrial compounds, natural solvents, and cleansing agents), water-soluble pesticides are causing great trouble. Poor management, particularly in on-farm handling, is the primary contributor to pesticide contamination in the ecosystem.^27^ There are many different sources of pesticide contamination, such as industrial manufacturing processes, the discharge from agricultural activities like container cleaning, spraying, and washing contaminated crops, soil degradation, atmospheric deposition, and many more, which potentially lead to environmental contamination through processes like bioaccumulation.^28^ So basically, the persistent pesticides reach the environment by either direct or indirect means. What's more, pesticide pollution originates from two main categories of sources: point sources (specific) and non-point sources, also known as diffuse sources. This includes scenarios like the pesticide's transfer from various surface water sources, leading to water pollution, which affects both aquatic and land ecosystems. Along with other sources, agriculture is a major contributor to diffuse pollution, which is caused by activities without specific release points.^29^ On a global and regional level, non-point source pollution is a major environmental challenge and is acknowledged as a major contributor to the deterioration of water quality. According to reports, non-point source pollution has affected between 30% and 50% of surface water bodies globally.^30^

Overview of pesticides and their environmental impact revealed that the excessive use of pesticides ultimately leads to their accumulation in soils, then either eliminates microorganisms that are essential for many biological processes, like nutrient uptake or organic matter breakdown, or inhibits their activity, which leads to lower soil fertility.^31^ These inadequately and excessively used products can also harm non-targeted organisms because only a small portion (1–3%) of the pesticides being used reach their targets. The dispersal of contaminants to non-target areas occurs irrespective of any natural barriers, and because of multiple transport pathways, it can be difficult to track the contamination.^32^ Preliminary findings show that aquatic organisms are seriously endangered due to the presence of pesticides in waterbodies. Fish communities experience behavioural and physiological changes, and aquatic plants suffer from lower oxygen levels in the water.^33^ Additionally, pesticides affect terrestrial fauna populations, including beneficial insects, whose numbers may significantly decrease when broad-spectrum insecticides are applied.^34^ Alternatively, the accumulation of pesticides in birds' and mammals' tissues has been responsible for a decline in their population because of the adverse effects on the nervous systems. It causes behavioural changes that can lead to death.^35^ Through contaminated feed, water, or direct touch, farm animals are also exposed to pesticides, which can have detrimental effects. Multiple studies report that acute toxicity, immune system weakness, reproductive issues, and organ damage are all possible outcomes of pesticide exposure in farm animals (Table 2). Even at low concentrations, prolonged exposure might cause long-term health problems such as hormone imbalances and decreased productivity. Pesticide residues that bioaccumulate in animal tissues can also endanger human health by way of the food chain.

**Table 2:** Pesticide impacts on farm animals

Pesticide class	Adverse effects in farm animals	Ref.
Carbamates	Neuromuscular weakness; potential residues in milk/eggs if misuse	43
Pyrethroids	Dermal/respiratory irritation; stress; resistance issues in stable flies affecting cattle	44
Neonicotinoids	Potential residue transfer *via* feed; limited direct farm-animal toxicity at labelled uses, but monitor feed contamination	45
Phenoxy herbicides	Indirect effects *via* forage contamination, and if misused, may lead to milk residue risks (*e.g.*, feed-to-milk pathway)	46
Organochlorine	Bioaccumulation in animals and milk, causing potential human exposure	47

**Fig. 2:**
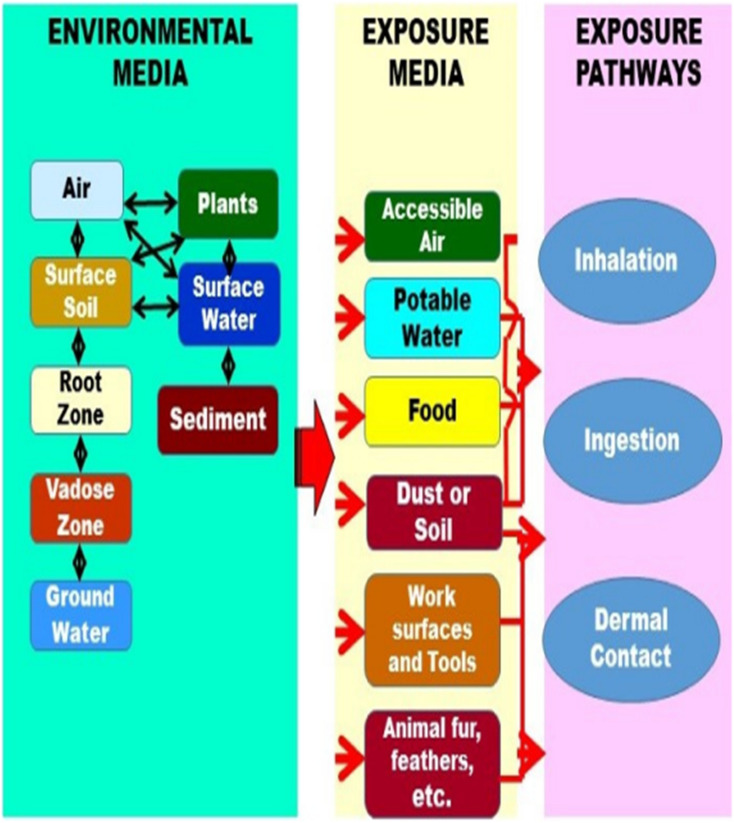
Pathways of pesticides entry into the human body.^15^ Reproduced from ref. 15 with permission from MDPI, copyright 2022.

In a similar manner, humans can be adversely affected by pesticides through direct exposure to them during agricultural activities (Fig. 2). Because pesticides came into contact with humans *via* household vegetation, or having occupational-related farming, as well as indirectly through the food chain and environmental contamination.^36^ Exposure of humans to pesticides conveys a significant health risk, with both acute effects and chronic effects on health. Numerous health consequences result from these effects, including acute and long-term neurotoxicity from fungicides, insecticides, and fumigants; lung damage from the pesticide paraquat; infant methemoglobinemia from nitrate seeping into groundwater; and chemical injuries like burns from pesticide exposure, such as anhydrous ammonia.^37^ Furthermore, a variety of cancers have been linked to pesticide exposure, including hematopoietic cancers, cancers of the digestive tract, cancers of the reproductive system, bladder cancer, breast cancer, and lung cancer.^38^ Cholinergic effects include the possibility of immunologic abnormalities^39^ and adverse effects on reproductive and developmental processes.^40^ Moreover, pesticides can have an impact on health even if a person is not showing any serious signs of illness. People who get exposure often report difficulty in localizing sensations while their muscle strength is reduced.^41^ The causes of diseases that may arise from exposure to pesticides can vary depending on a number of factors. These variables include the kind of pesticide applied, the exposure method and duration, and the general health of the person. Pesticides undergo various processes after entering the bodies of animals or humans, which include metabolism, excretion, storage, and accumulation in adipose tissues^42^ as shown in Table 3.

**Table 3:** Main classes of pesticide and their effect on human health

Pesticide class	Effect	Ref.
Organophosphate	Chronic central nervous system disorders, respiratory disorders, cardiovascular diseases, diabetes mellitus, cancer, infertility issues, impaired vision, Alzheimer's disease, cellular oxidative stress, Parkinson's disease, hypotension, *etc.*	48–50
Organochlorine	Cardiovascular disorders, hypertension, neurological disorders, hormone-related cancers (breast, prostate, lung, stomach), obesity, endocrine disruption, alterations in embryonic development, hematologic and hepatic changes, diabetes in overweight individuals, learning disabilities in children, hyperactivity disorder, and Parkinson's disease	51 and 52
Carbamate	Immunotoxicity, cholinergic poisoning, male infertility, rndocrine disruption and inhibition of esterases, endoplasmic reticulum stress, *etc.*	53 and 54
Pyrethroids	Respiratory distress, nausea, tachycardia, apathy, metabolic acidosis, convulsions, anaphylactic shock, pulmonary edema, and oxidative stress. Risks to reproductive health and neurobehavioral, cancer, and the development of autism spectrum disorders in infants, *etc.*	55–57

## 3. Conventional method of pesticide remediation

**Fig. 3:**
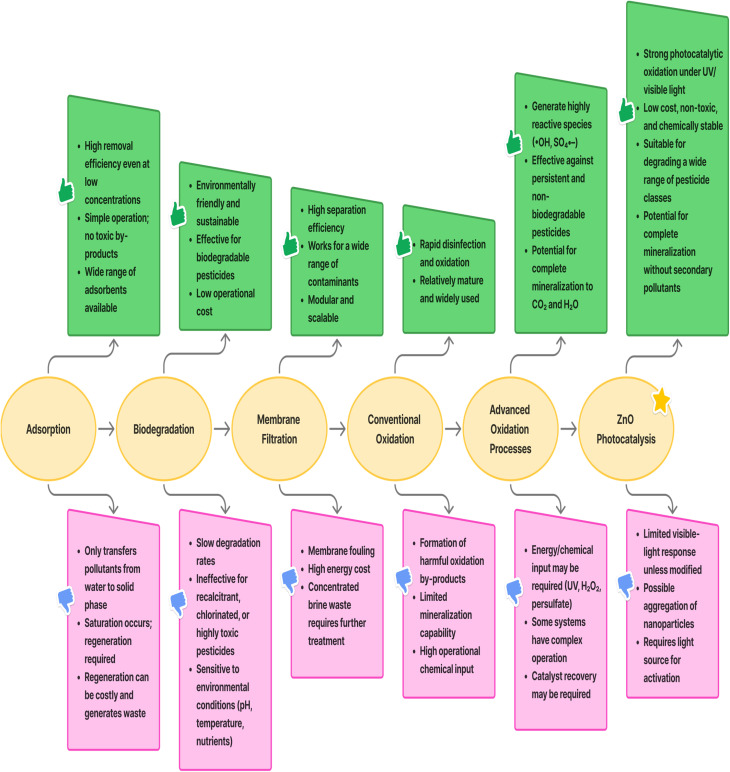
Comparison of treatment methods for pesticide removal: distinct advantages and limitations associated with each approach.

Currently, various remediation methods are utilised for addressing the water^58^ and soil^59^ contamination with pesticides. If one technology proves ineffective, combining multiple methods becomes necessary to achieve satisfactory outcomes. Therefore, numerous techniques have been suggested and implemented over time to eliminate persistent pollutants. Traditional approaches for eliminating these pollutants include membrane filtration,^60^ surface trapping,^61^ ozonolysis, air stripping, skimming, photolysis, and Fenton oxidation processes.^62^ Biodegradation, particularly through microbial action, is also found to be a versatile and effective strategy for remediating pesticide-contaminated sites due to the capability of microbes to function even in harsh environmental conditions.^63^ However, this method is both time-consuming and inefficient, with limitations in its applicability. Likewise, processes like sedimentation, membrane technologies, and chemical filtration entail significant operational expenses and result in the generation of highly poisonous secondary pollutants that enter nature.^64^ In addition, various physicochemical techniques such as reverse osmosis, carbon adsorption, adsorption, nano-filtration, distillation, adsorption, and ion exchange resins have also been utilised recently. Adsorption and coagulation are the processes that primarily concentrate pollutants, changing their phase, rather than eliminating or degrading them completely.^65^ Therefore, these methods encounter significant challenges, including issues with disposal, membrane distortion, formation of sludge, operational handling, and various technical limitations.^66^ In summary, the various physical, chemical, and biological methods employed for pesticide removal each possess distinct advantages and disadvantages. These characteristics influence their effectiveness based on factors such as the type of pollutant, its concentration, and the specific goals of the treatment, as illustrated in Fig. 3.

Since the majority of persistent pollutants are resistant to breakdown through conventional chemical and biological treatment approaches. The methods described above are efficient, but they have limitations in terms of their effectiveness, applicability, cost, and time. Consequently, the inadequacy of current treatment processes for pesticide remediation has heightened the need for advanced technologies for treatment. So, scientists have explored alternative approaches to degrade persistent pesticides completely into eco-friendly substances, such as the advanced oxidation process (AOPs).

## 4. Advanced oxidation process

To address the downsides of traditional treatment methods, scientists are exploring AOPs as a more effective way to remove pesticides from wastewater. AOP stands out as an exceptionally effective and advantageous method for purifying polluted water. It efficiently transforms pesticides or any other organic contaminants into water, carbon dioxide, and basic salts.^67^ It employs a potent oxidant like hydroxyl radical (OH˙), possessing the second-highest oxidising power, approximately 2.8 eV less than fluorine. These radicals can interact with nearly all organic pollutants at rate constants ranging from 10^6^ to 10^9^ mol L^−1^ s^−1^.^68^ This advanced technology is becoming more promising and an increasingly adopted method for addressing recalcitrant wastewater, containing a lot of organic matter and low pH levels. Moreover, this approach is employed to neutralise pathogens after secondary treatment.^69^

**Fig. 4:**
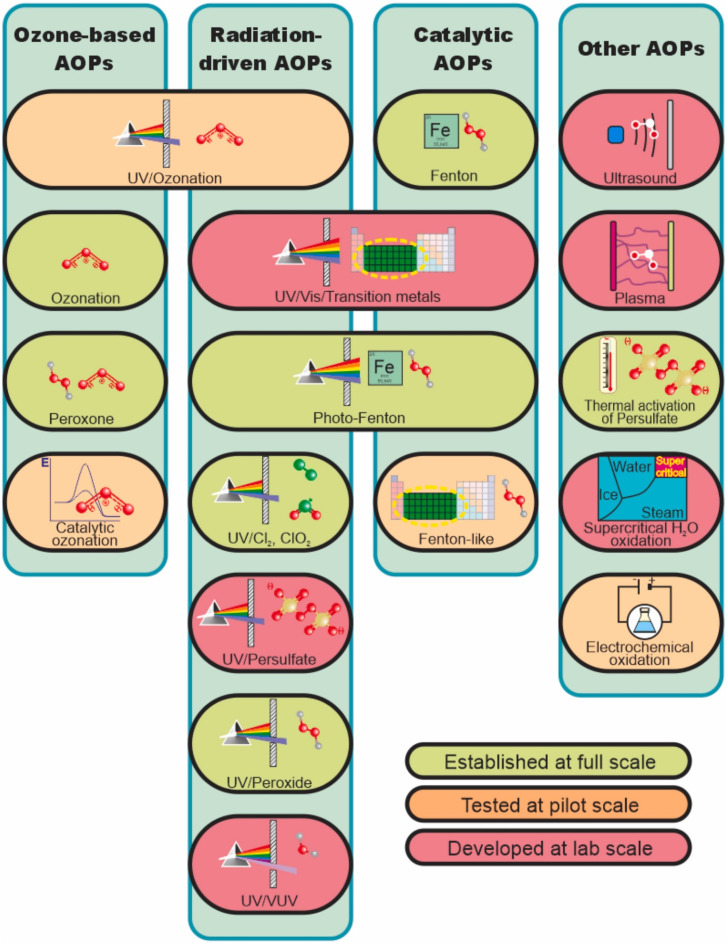
Classification of AOPs. This figure has been reproduced with permission from ref. 73, Elsevier, copyright 2024.

Generally, AOP involves activating semiconductor photocatalysts through light that leads to the formation of OH˙ that oxidizes pollutants. The reduction of adsorbed oxygen molecules generates oxygen radicals that help break down pollutants.^70^ The indiscriminate characteristic of OH˙ renders them suitable for remediation in the environment.^71^ Additional oxidative agents employed in AOP include ozone and superoxide radicals (O^2−^).^72^ The general classification of AOP can be seen in Fig. 4.

## 5. Photocatalysis

**Fig. 5:**
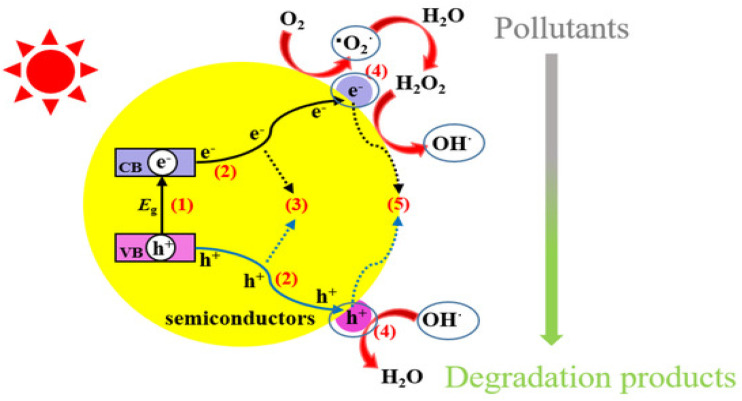
Photocatalytic processes over a heterogeneous photocatalyst.^79^ Reproduced from ref. 79 with permission from MDPI, copyright 2021.

Within this sequence of procedures, photocatalysis, which relies on the use of a photocatalyst to absorb accelerated photons, is one of the most effective methods in the AOP. According to the Photocatalysis Industry Association of Japan, a photocatalyst is a substance that accelerates chemical reactions by utilizing an external energy source. One important application of photocatalysts is the degradation of harmful substances such as pesticides. By generating reactive free radicals, particularly OH˙, photocatalysts can convert highly toxic pesticides into less harmful compounds.^74^ This process begins when the photocatalyst absorbs radiation with wavelengths longer than 290 nm. The absorbed energy excites electrons in the catalyst material, promoting them to a higher energy state. These excited electrons, along with the resulting positive “holes” left behind, can interact with surrounding molecules. The interaction leads to the formation of reactive species such as superoxide and OH˙. These radicals then react with the active components of pesticides or other pollutants through oxidation–reduction reactions (Fig. 5), ultimately leading to their breakdown and the detoxification of water.^75^

Photocatalysts offer safety, cost-effectiveness, exceptional durability, chemical and biological inertness, insolubility in most scenarios, and the ability to be recycled and reused. They can be activated by both artificial light and sunlight.^76^ However, there are several challenges in photocatalysis research that must be addressed before practical application. These include the inability to function effectively in solar radiation, issues with reactor design, limitations on catalyst reuse and recovery, lower quantum efficiency, and the potential production of toxic byproducts.^77^ Another challenge in photocatalysis is the rapid recombination of photo-generated electron–hole pairs, which can hinder the efficiency of the process. This phenomenon, known as charge recombination, results in the release of electron energy as heat, ultimately terminating the reaction. To address this issue, doping with metal oxide nanomaterials is recommended. Doping helps prevent electron–hole recombination by introducing metal ions, thus extending the lifespan of electron–hole pairs enough to facilitate redox reactions at the catalyst surface.^78^

The photocatalysis process is of two kinds: homogeneous and heterogeneous. Homogeneous photocatalysis occurs when a soluble catalyst and reactant are present in a single phase. The derived homogeneous solution contains photon promoters and active catalytic sites that function in two different ways. In the first technique, a photosensitizer is used to transfer electrons to the catalyst, creating an active site that triggers a reduction reaction. In the second technique, the catalyst acts as both a catalyst and a substance that absorbs light. When light incidence excites the catalyst electrons, they shift from the highest occupied molecular orbital to the lowest unoccupied molecular orbital, causing the catalyst to act as a good substance for redox processes.^80^ Transition metal complexes are the most widely used homogeneous photocatalyst due to their advantageous energy band gap properties and stability, while ozone is the second most commonly used catalyst.^70,81^ The main disadvantage of a homogeneous photocatalyst system is that it is difficult to separate the photocatalyst from the solution since it is completely soluble.^82^

Heterogeneous photocatalysis refers to the enhancement of photoreactions in the presence of a catalyst.^83^ The reaction involves the interaction of substances with multiple states. Typically, a solid photocatalyst is used, and organic pollutants or compounds in the aqueous phase are exposed to the catalyst's surface for photo degradation.^84^ Commonly used materials in heterogeneous photocatalysis include ZnO, ZnS, MnO_2_, WO_3_, MoO_3_, TiO_2_, SnO_2_, Fe_2_O_3_, CdS, CeO_2_, and ZrO_2_ (Table 4). These materials are favoured due to their chemical and mechanical stability, as well as their inertness towards biological tissues. They have a low energy gap and possess properties like high porosity, large surface area, and both hydrophobic and hydrophilic interactions that make them well-suited for the photodegradation of persistent pesticides.^85,86^ Heterogeneous photocatalysis is a complex process involving multiple stages to eliminate harmful substances from wastewaters. Generally, the process starts when light of sufficient energy strikes the catalyst's surface, and it is absorbed, causing an electron to move from the valence band (VB) to the conduction band (CB), leaving a hole in the valence band. This process generates an electron–hole pair called an exciton. A critical aspect of heterogeneous photocatalysis is to prevent the recombination of these electron–hole pairs and utilize excitons for a redox reaction to fully degrade toxic compounds into harmless minerals.^87^

**Table 4:** Band gap energies of a semiconductor

Semiconductor	Band gap (eV)	Ref.
ZnO	3.37	88
TiO	3.20	89
MnO_2_	1.38 or 1.41	90
Fe_2_O_3_	1.77 to 2.25	91
SnO_2_	3.66	92

Nanomaterials, which are known for their small size and unique properties, have emerged as a promising area of interest for effectively addressing various environmental pollutants, including organic pesticides. A diverse array of materials, including metal oxides (such as TiO_2_, ZnO, CuO, MgO), metal nanoparticles (like Au, Ag), bimetallic nanoparticles, bio-nanopolymers (such as alginate–Ag, ZnO–cellulose), adsorbents (including triggered charcoal, zeolites, calcite, clays, and other carbonaceous materials), as well as nanoparticles and nanocomposites, have been extensively utilized for pesticide remediation over decade.^93^

**Fig. 6:**
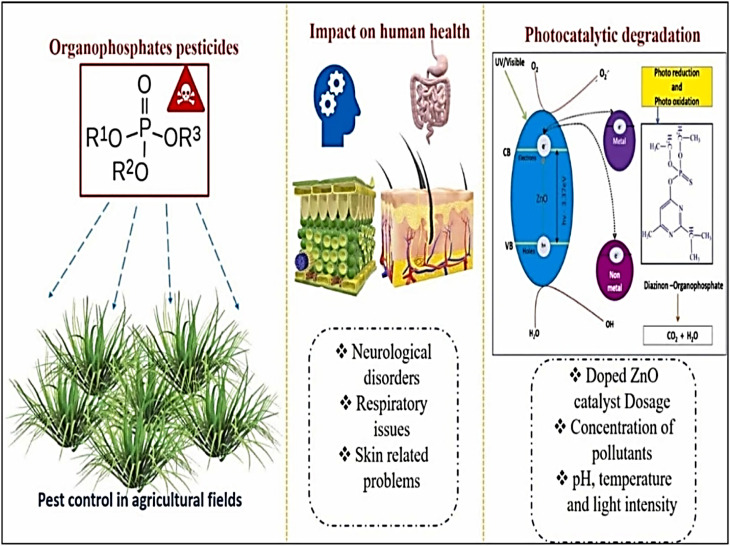
Photocatalytic degradation of the health hazardous organophosphorus pesticides using ZnO coupled photocatalysts. This figure has been reproduced with permission from ref. 95, Elsevier, copyright 2024.

Typically, nano-based remediation technology utilizing nanomaterials as photocatalysts stands out as the most effective advanced oxidation method for addressing pesticides and further harmful pollutants. By using photo-excitation, the photocatalyst creates both electron-donating (reducing) and electron-accepting (oxidizing) species (Fig. 6), which have great potential as redox agents. Technology like nano-photocatalysis follows the principles of green chemistry to remove harmful pollutants from the environment and human life. It achieves this by breaking down stubborn compounds (Fig. 6) into intermediary substances and eventually into harmless byproducts.^94^

## 6. ZnO and its nanomaterials as photocatalysts

Since visible light makes up the bulk of the solar spectrum, there is a rising need to use solar energy to combat water pollution, which has led to an increased focus on Visible Light Active (VLA) photocatalysts. An ideal photocatalyst should function under both UV and visible light, remain chemically stable, resist photo-corrosion, be environmentally safe, and remain cost-effective,^96^ but to do so, factors such as surface area, porosity, crystalline structure, particle size, and band gap play a critical role.^66^

ZnO is classified as a II–VI semiconductor, characterized by a direct band gap of approximately 3.37 eV and an exciton binding energy of around 60 meV. It adopts a wurtzite hexagonal crystal structure, where the tetrahedral coordination of Zn^2+^ and O^2−^ ions leads to significant polarization, influencing its electronic band structure. The conduction band is primarily derived from the Zn 4s orbitals, while the valence band is formed from the O 2p orbitals. When exposed to UV light, ZnO facilitates the generation of electron–hole pairs, as illustrated in eqn (1):

1 ZnO + ℏ*ν* → e_CB_^−^ + h_VB_^+^

In nanoscale ZnO, the phenomena of quantum confinement, the presence of surface states, and oxygen vacancies contribute to the formation of shallow donor levels. These factors enhance electron density, facilitate stronger adsorption, and promote efficient electron transfer to pesticide molecules. Additionally, when ZnO is synthesized using environmentally friendly or waste-derived methods, it aligns with green chemistry principles by reducing resource consumption and minimizing its environmental impact.

**Fig. 7:**
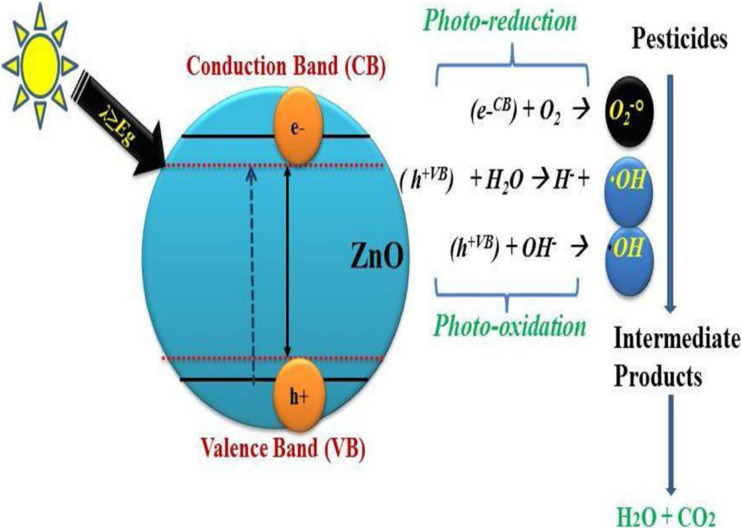
Photocatalytic degradation mechanism at the surface of ZnO. This figure has been reprinted with permission from ref. 97, Elsevier, copyright 2020.

ZnO exhibits band edges of approximately −0.5 eV for the conduction band and +2.7 eV for the valence band relative to the NHE, facilitating the generation of both superoxide (˙O_2_^−^) and hydroxyl radicals (˙OH). Under UV light excitation with wavelengths shorter than 380 nm, electrons are able to reduce O_2_ to ˙O_2_^−^, while the holes oxidize water or OH^−^ to yield ˙OH, as depicted in Fig. 7. Reactive oxygen species exhibit a potent oxidative capacity, enabling them to break down pesticides, dyes, pharmaceuticals, and other enduring pollutants into benign products such as CO_2_ and H_2_O.^97^ The photocatalytic performance of ZnO is strongly influenced by band gap energy, charge carrier dynamics, and synthesis-dependent morphological factors.^98^

Similarly, at the surface of ZnO nanoparticles, upon visible light interaction, mechanistic changes start. The photoreaction begins when electron–hole pairs are generated that migrate across the nanoparticle surface. These pairs undergo oxidation and reduction reactions on the surface of the catalyst. Upon contact with water molecules, the holes produce hydroxyl ions (OH^−^) and convert into hydroxyl radicals OH˙. Electrons react with molecular oxygen to form superoxide ions (O_2_˙^−^), which can further react with hydrogen ions to produce hydrogen peroxide (H_2_O_2_). Superoxide and H_2_O_2_ radicals then combine to create more OH˙ (potent oxidizing agents that break down organic pollutants into harmless products).^99^ These ROS, along with photogenerated holes, attack the organic pollutants, forming oxidized intermediates that undergo fragmentation toward partial or complete mineralization (CO_2_, H_2_O, and inorganic ions). The process of heterogeneous photocatalysis involving ZnO as the catalyst operates as follows (eqn (2)–(12)).^70,100^

2 ZnO + ℏ*ν* → ZnO(e^−^) + ZnO(h^+^)

3 ZnO[h^+^(VB)] + H_2_O → ZnO + H^+^ + OH˙

4 ZnO[h^+^(VB)] + OH^−^ → ZnO + OH˙

5 ZnO[e^−^(CB)] + O_2_ → ZnO + O_2_˙^−^

6
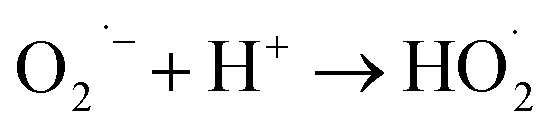
7
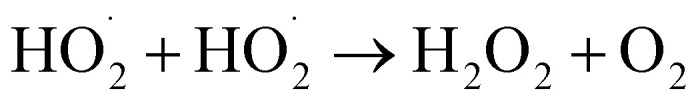

8 ZnO[e^−^(CB)] + H_2_O_2_ → OH˙ + OH^−^

9 H_2_O_2_ + O_2_˙^−^ → OH˙ +OH˙ + O_2_

10 H_2_O_2_ + ℏ*ν* → 2OH˙

11 Organic pollutants + OH˙ → intermediates

12 Intermediates → CO_2_ + H_2_O

In case of pesticides remediation using ZnO, the pesticides undergo photolysis, a process in which UV-visible light breaks down their large molecules into smaller and simpler ones. During this process, photons are absorbed by pesticide molecules or surrounding species (such as water or sensitizers), leading to bond cleavage. For example, when water absorbs high-energy photons, it can undergo photolysis: H_2_O + ℏ*ν* → ˙OH + H˙. These highly reactive ˙OH and H˙ radicals can then attack pesticide molecules, leading to their degradation.^101^ The fundamental idea behind photocatalysts is their ability to generate electron–hole pairs, which is how OH˙ are created.^102^

Research has increasingly favored ZnO over other photocatalysts for pesticide degradation, particularly when compared to titanium dioxide (TiO_2_), which has been the most extensively studied photocatalyst for contaminant removal. Despite its popularity, TiO_2_ has notable drawbacks, such as low quantum efficiency, rapid charge recombination, and a wide band gap of approximately 3.2 eV, which limits its activation to ultraviolet light. These limitations have prompted a shift in focus toward ZnO as a more effective alternative. With comparable photocatalytic activity and improved UV absorption, ZnO also allows easier modification for visible-light activity.^103^ ZnO further distinguishes itself with superior biological, optical, catalytic, photochemical, and antibacterial properties compared to TiO_2_, and has shown enhanced photocatalytic efficiency in dye-contaminated aqueous systems.^104^ Its higher electron mobility (two orders of magnitude greater than TiO_2_),^105^ together with versatile synthesis routes enabling diverse morphologies,^106^ enhances its applicability. The unique properties of ZnO, including its band structure, wide band gap, highly positive valence band, and the presence of abundant defects, contribute to its effectiveness in degrading persistent pesticides. Additionally, its strong ROS generation and high charge mobility, coupled with its low cost and non-toxic nature, position ZnO as a superior photocatalyst compared to many alternatives, as listed in Table 5. Unlike traditional treatment methods, ZnO photocatalysts stand out because they do not produce secondary pollutants, require no external chemicals and operate without high energy inputs. Their capacity to harness natural sunlight renders the process low-carbon and particularly suitable for decentralized or rural wastewater systems, where energy resources may be limited.

**Table 5:** Comparative analysis of ZnO with other photocatalysts: pros and cons for pesticides degradation

Photocatalyst	Advantages of ZnO	Disadvantages of ZnO	Ref.
ZnO *vs.* TiO_2_	• Higher quantum efficiency	• TiO_2_ is more stable under acidic/basic conditions	107
	• More oxygen vacancies (stronger ROS)	• ZnO can undergo photocorrosion	
	• Stronger oxidative holes (higher valence band)		
	• Better adsorption of polar pesticides		
	• Faster electron mobility		
ZnO *vs.* Fe_2_O_3_	• Fe_2_O_3_ has very poor charge mobility	• Fe_2_O_3_ is magnetically recoverable	108
	• ZnO has faster electron transfer		
	• Fe_2_O_3_ absorbs visible light but produces very weak ROS		
ZnO *vs.* spinel ferrites (ZnFe_2_O_4_, CoFe_2_O_4_)	• Ferrites have extremely high recombination	• Ferrites are magnetically recoverable	109
	• ZnO produces stronger ˙OH		
	• Ferrites often require composites to work well		
ZnO *vs.* g-C_3_N_4_	• g-C_3_N_4_ alone is weak under UV	• g-C_3_N_4_ works better under visible light alone	110
	• ZnO produces stronger oxidative holes	• ZnO needs UV or doping to shift into the visible region	
	• ZnO adsorbs pesticide molecules more strongly (especially polar pesticides)		
ZnO *vs.* WO_3_	• WO_3_ has a very low conduction band (cannot generate ˙O_2_^−^ radicals)	• WO_3_ is more stable in acidic conditions	111
	• ZnO can produce both ˙OH and ˙O_2_^−^ which leads to more complete pesticide mineralization		
	• ZnO offers a higher degradation rate for chlorinated pesticides		
ZnO *vs.* Ag-based photocatalysts	• ZnO is cheap, non-toxic	• Ag-based catalysts can show better visible-light activity	112
	• Ag is expensive and toxic, risk of Ag^+^ leaching		
	• Ag nanoparticles can deactivate due to oxidation		

## 7. Methods to enhance photocatalytic properties of ZnO and its nanomaterials

The photocatalytic performance of ZnO nanoparticles is determined by their synthesis technique. The hydrothermal and sol–gel techniques produce extremely crystalline, uniform ZnO with great efficiency but require longer processing durations. Chemical precipitation is less expensive and more environmentally friendly than solvothermal synthesis, which generates high-purity ZnO but requires careful control and a lot of energy. Green synthesis provides an environmentally friendly alternative with middling efficiency (Table 6). Other approaches, including microwave-assisted, electrochemical, sonochemical, spray pyrolysis, and combustion, offer greater versatility by altering particle size, crystallinity, and overall photocatalytic activity.

**Table 6:** Influence of synthesis approach on the photocatalytic activity of ZnO nanomaterials

Method	Photo-catalyst	Band gap (eV)	Efficiency	Advantage/limitation	Ref.
Hydrothermal	ZnO NPs, nitrogen-doped ZnO	3.19	99.60	High crystallinity and purity, but requires extended processing times	113 and 114
Sol–gel	Nanosized ZnO	3.30	99.00	Simple and adaptable; produces uniform materials but may take longer to dry	115 and 116
Chemical precipitation	Nanosized ZnO	3.20	97.36	Low-cost, eco-friendly, and efficient; produces highly active ZnO photocatalysts	117
Solvothermal	Nanosized ZnO	2.99	92.00	The solvothermal method produces high-purity, uniform ZnO photocatalysts but demands high energy, long reaction times, and precise control	118 and 119
Green synthesis	Nanosized ZnO	3.20	87.00	Environmentally friendly, low-cost, and highly efficient; variability in quality may occur depending on the plant source	120

However, synthesized ZnO still faces several intrinsic limitations that hinder its photocatalytic efficiency. One of the primary challenges is the rapid recombination of photogenerated electron–hole pairs, which significantly reduces the number of charge carriers reaching the catalyst surface. This recombination not only slows down the redox reactions but also leads to energy loss in the form of heat. Additionally, ZnO's wide band gap restricts its light absorption to the UV region, limiting its effectiveness under visible light irradiation.^9^ Another drawback is the tendency of ZnO nanoparticles to agglomerate, which decreases the effective surface area available for light absorption and reactive species generation. These larger aggregates also reduce the photon flux within the reaction medium, further lowering the photocatalytic activity. Moreover, charge carrier recombination within these aggregates contributes to a significant decline in overall performance.

To overcome these limitations, various strategies have been developed to enhance the photocatalytic performance of ZnO. These include modifying its physical structure to increase surface area, doping with noble or non-noble metals to alter electronic properties, and forming heterojunctions with other semiconductors to promote charge separation. The deliberate introduction of structural defects has also been shown to improve light absorption and trap charge carriers.^121^ Additionally, coupling ZnO with plasmonic and photothermal materials can extend its light absorption range into the visible spectrum and enhance photocatalytic efficiency through localized surface plasmon resonance.^122^

### 7.1. Doping to modify photocatalysts

#### 7.1.1. Doping with metal

Various transition metals such as silver (Ag), manganese (Mn), nickel (Ni), copper (Cu), and iron (Fe) have been utilized as dopants in ZnO; however, studies have shown that only optimal concentrations result can enhanced photocatalytic activity.^123^ By reducing the band gap, doping with metal enables ZnO to absorb visible light, which makes up 40–45% of sunlight. Furthermore, doping modifies the photocatalyst's optical and electrical characteristics.^124^ Consequently, ZnO doped with different metals is used in a variety of industries, such as paints, chemicals, tires, ceramics, pharmaceuticals, and agriculture. For example, scientists investigated the removal of chromium(vi) using a biosynthesized Ag-doped ZnO nanocomposite that contained activated carbon. After 60 hours of treatment, they discovered that the optimal adsorption took place at pH 2.5, with a concentration of 40 ppm of heavy metal ions.^125^ When Fe^3+^ ions are added to ZnO nanoparticles, more Zn^2+^ is produced, which increases surface defects and improves degradation performance.^126^ Ag-doped ZnO nanoparticles also show that noticeable surface defects improve degradation performance.^127^ Additional finding also suggests that aluminum (Al) increases hydrophilicity and surface defects in ZnO while cathodically shifting the quasi-Fermi level. By creating smaller particles, Al doping also increases specific surface area, which promotes the breakdown of organic pollutants.^128^ According to a study, hydroxyl ion absorption on nanoparticle surfaces is increased when high concentrations of magnesium (Mg) dopant are added to ZnO. As a result, carriers are efficiently trapped by abundant Mg^2+^ ions, which lowers recombination and speeds up degradation.^129^ At room temperature, Cu-doping with ZnO increases the effectiveness of both photocatalytic and antioxidant activities. These nanoparticles are also versatile for wastewater treatment applications due to their pH-dependent photocatalytic properties.^130^ It has been demonstrated that doping ZnO photocatalyst with rare earth metals, such as lanthanum (La), increases its efficiency. A study using La-doped ZnO photocatalysts to degrade 2-chlorophenol (2-CP) found that the degradation efficiency was higher when the catalyst dose was 10 mg and the irradiation period was 2 hours at an ideal pH of 2.^131^Table 7 contains the list of some metal dopants that are used with ZnO for photocatalytic applications.

**Table 7:** Impact of metal dopants on improving the photodegradation capabilities of ZnO

Doping metal	Efficiency of ZnO (%)	Efficiency of doped-ZnO (%)	Contaminant degraded	Ref.
Ag	90.00	98.00	Carbaryl	132
La	75.85	83.92	2-Chlorophenol	131
Cu	58.50	96.97	Diazinon	133
Pd	38.00	82.00	Acetamiprid	134
Pd + graphene oxide	38.00	98.00		
WO_3_	27.00	78.00	2,4-Dichlorophenoxyacetic acid	135

#### 7.1.2. Doping with non-metal

Non-metal dopants such as carbon (C), nitrogen (N), fluorine (F), iodine (I), and sulphur (S) improve ZnO composite photocatalytic efficiency under visible light. They modify the bandgap by replacing oxygen vacancies, increasing surface oxygen vacancy defects. Their minute size enables diffusion through lattice interstices, binding to atoms *via* oxidation to aid the process.^136^ In contrast to metal ion dopants, non-metal ions have a lower likelihood of forming recombination centres. Therefore, they are more effective in enhancing photocatalytic activities. In the same context,^137^ explored the impact of N-doping on ZnO nanoparticles for degrading methylene blue dye, and observed superior efficacy compared to pure ZnO under both UV and visible light. The author explains how N-doping lowered excitation energy, creating new energy states near ZnO's VB, thereby increasing electron–hole pair production under visible light. This narrower band gap facilitated more straightforward electron transfer and intensified oxygen vacancies, facilitating rapid carrier separation and enhancing photocatalytic activity over three cycles. In a separate study,^138^ synthesized N-doped ZnO composites using a sol–gel combustion method, demonstrating excellent performance in degrading Eosin Yellow under visible light. XPS analysis confirmed the formation of N–Zn bonds, while improved photo response in the visible region resulted from N-induced lattice defects. Catalytic activity was increased by the composite's high surface area and porosity, which allowed for improved dye-catalyst contact. Likewise,^139^ investigated the photocatalytic degradation of *p*-aminobenzoic acid using C-doped ZnO nanorods as a catalyst. A cost-effective precipitation method was used to produce nanorods. When exposed to sunlight, 97% of the *p*-aminobenzoic acid was degraded under ideal circumstances (0.5 g per L catalyst dosage). The nanorods maintained their high photodegradation efficiency and showed signs of reusability.

In another analysis,^140^ investigated I-modified ZnO's antibacterial qualities in the presence of light. With its cage-like structure and abundance of oxygen defects, the composite efficiently separated photoexcited charge carriers to produce more free radicals. Furthermore, iodine and the cage structure decreased electron–hole pair recombination, and photocatalytic performance was greatly enhanced by a smaller grain size. Additionally, by using differently shaped S-doped and F-doped ZnO,^141^ the photocatalytic degradation of methylene blue was compared. Under visible-light photocatalysis, these dopants changed the electron mobility rate and band gap structure. After six hours of exposure to visible light, S-doped ZnO showed better photoactivity (total removal) than F-doped ZnO. This was because S-doped ZnO had a smaller band gap and was more efficient at absorbing solar energy, which inhibited electron–hole recombination and increased photosensitive activity.

### 7.2. ZnO composites

Pairing ZnO with semiconductors can significantly boost its photocatalytic activity. This approach creates nanocomposites that improve light absorption, reduce recombination of charge carriers, and enhance charge separation. Combining ZnO with semiconductors that have different band gaps has shown great potential for improving photocatalysis.^121^ Ref. 142 studied that longer-lived charge carriers in these composites lead to more effective photo-degradation. While^143^ showed that adding CdS to ZnO changes its growth pattern, resulting in flower-like structures. The defects in the ZnO–CdS composite trap charge carriers, which reduce recombination and significantly enhance its ability to degrade rhodamine B (RhB).

Corresponding ref. 144 compared the degradation rate of chlorpyrifos, using MoO_3_/Al_2_O_3_ and ZnO·MoO_3_/Al_2_O_3_. The degradation kinetics showed that ZnO·MoO_3_/Al_2_O_3_ (95%) degraded chlorpyrifos 8 times faster than MoO_3_/Al_2_O_3_ (86%). In addition, intermediate products of chlorpyrifos were not identified by GC/MS analysis, which could be more toxic than chlorpyrifos itself, indicating a thorough mineralization of the pesticide,^145^ demonstrated that Fe–ZnO nanocomposite achieved 93.5% degradation of chlorpyrifos in 60 minutes under UV light. The enhanced performance is attributed to its reduced optical band gap and magnetic properties, making it effective for visible light photocatalysis. According to ref. 146, the β-CD–CuO/ZnO nanocomposite exhibits improved photocatalytic efficiency, degrading methylene blue by up to 89.15% and malachite green by 79.90% in 180 minutes when exposed to radiation. Its capacity to encapsulate pollutants in its hydrophobic interior and enhanced electron–hole recombination are the reasons for its superior performance.

Another nanocomposite, ZnO/BaBi_2_O_6_, demonstrated noticeably greater rates of methyl orange, RhB, and eosin degradation than pure ZnO, and it was able to degrade methyl orange by over 95% in 40 minutes. With a band gap energy of 2.89 eV and stability after five reuse cycles, h^+^ and radical O^2−^ were found to be important components in the degradation process.^147^ A sustainable biogenic process was used to create the ZnO/multi-walled carbon nanotubes composite, which exhibits remarkable photocatalytic efficiency in eliminating hazardous pollutants and has the potential to be used for environmental remediation with high reusability.^148^ In another study,^149^ chemical precipitation was used, which creates free radicals at the conduction band (superoxide ion) and valence band (hydroxyl group) levels in Ag/Fe_2_O_3_/ZnO heterostructure, producing effective photocatalysis and superhydrophobic qualities. In another study, Cu-MOF/ZnO nanocomposite was found to exhibit high photocatalytic efficiency, degrading rose bengal by 97.4% in 45 minutes when exposed to natural sunlight. It is a promising material for environmental remediation because it retains stability and effective performance for up to five reuse cycles.^150^

### 7.3. Impacts of structural defect

The structure and form of ZnO, or morphology, have a significant impact on its photocatalytic capabilities. For example, although rods have a larger surface area, researchers have found that hexagonal or spherical ZnO nanoparticles often perform better than rod-shaped ones.^151^ In other cases, researchers have also emphasized the enhancement of the performance of needle-shaped^152^ or nanowire ZnO.^153^ Moreover, studies have also been examining structural flaws in addition to the morphology of ZnO nanoparticles. In particular, oxygen defects have emerged as a major focus of current photocatalysis research.^154^

In this context,^155^ investigated ZnO nanoparticles and how structural defects influence their photocatalytic efficiency in degrading RhB dye under UV light. Their findings revealed that the catalyst with the highest number of defects exhibited the greatest degradation performance. This improvement was attributed to an increased specific surface area resulting from the presence of more defects. Research suggests that zinc and oxygen vacancies can act as traps for charge carriers, which helps prevent the recombination of electron–hole pairs and thus enhances photocatalytic activity. For instance, ultrathin ZnO/Al_2_O_3_ nanosheets with a high density of oxygen defects were shown to rapidly degrade tetracycline and RhB dye within 150 minutes, achieving degradation rates of 88.4% and 76.9%, respectively.^156^ Similarly,^157^ synthesized ZnO/NiO nanoparticles with oxygen vacancies using solvothermal and high-temperature reduction methods to enhance their photocatalytic performance. The best solar-driven photocatalytic performance was shown by OZN-10, which almost degraded methylene blue due to its small size and large surface area. Because of its distinct structure and surface flaws, its efficiency was roughly double that of pure ZnO.

Ar–ZnO, which had the highest concentration of oxygen vacancies, showed a reduced bandgap of 3.03 eV, according to an experiment conducted by ref. 158 in which flower-like ZnO photocatalysts with porous nanosheets were prepared under various calcination atmospheres. This enhancement resulted in a 94.5% degradation of methyl orange under UV light in 30 minutes, with a degradation rate constant 3.2 times higher than ZnO calcined in air. Moreover, defect engineering in ZnO ceramics improved electrical conductivity and reduced thermal conductivity, enhancing their thermoelectric performance by increasing oxygen vacancies.^159^ Oxygen defects are known to boost photocatalytic efficiency and indicate that more defects lead to a larger surface area. However, some research suggests that oxygen and zinc defects can sometimes negatively affect ZnO's photocatalytic performance by serving as recombination centres, which reduces its effectiveness.^98^

## 8. Remediation of pesticides by ZnO photocatalysts

Over the years, there has been significant focus on utilizing ZnO and its nanoparticles as photocatalysts to break down pesticides. The majority of research on ZnO as a catalyst indicates that its photocatalytic efficacy in aqueous environments is heavily influenced by factors such as light source and intensity, reaction conditions, catalyst type, presence of oxidizing agents, solution pH, temperature, and pesticide concentration.^160^ Ref. 161 investigated the impact of the synthesis medium (ethanol and water) on the efficiency of ZnO/carbon xerogel photocatalysts to degrade 4-chlorophenol and bisphenol A. Carbon xerogel was chosen due to its electrical conductivity, surface area, and porosity. The maximum degradation rates achieved were 88% for 4-chlorophenol and 78% for bisphenol A after 5 hours. The photocatalytic mechanism relies heavily on the generation of OH˙, and the materials remained stable for up to three reuse cycles. The result shows that hybrid systems have shown greater efficiency in recent years when compared to pure systems. Another study^162^ shows that the GO–ZnO nanocomposite is excellent for breaking down the organophosphate pesticide quinalphos in water when exposed to UV light. This approach performs better than graphene GO nanosheets and ZnO nanoflowers. The nanocomposite exhibits pseudo-first-order kinetics and reaches a 98% degradation rate in 45 minutes at pH 6. Researchers found that OH˙ were the main active species in the degradation process after identifying smaller, innocuous byproducts through LC-MS analysis. The produced nanocomposite offers a workable way to degrade pesticides without requiring neutralization before being released into water bodies because it is stable and reusable for at least five cycles. Similarly,^163^ found that fungicide residues (difenoconazole and thifluzamide) in soil samples can be efficiently removed by chitosan–ZnO nanoparticles, which remain for days in the absence of these nanoparticles. In contrast, no activity was seen with chitosan–ZnO nanoparticle; photocatalytic studies demonstrated a significant increase in activity over a predetermined period. This indicates promising environmental remediation solutions and emphasizes the critical role that chitosan–ZnO nanoparticles play in promoting pesticide degradation.

Ref. 164 discovered that the pesticide chlorpyrifos is efficiently degraded by Ni-doped ZnO–TiO_2_ nanocomposites. These nanocomposites have a large surface area, distinct crystallinity, and good optical qualities. They convert chlorpyrifos into innocuous byproducts and function well in both visible and ultraviolet light. When exposed to UV light instead of darkness, the electrochemical analysis performed better. Degradation proceeds according to pseudo-first-order kinetics, with UV light causing higher rates (0.0221 min^−1^) than visible light (0.0088). In related work^165^ using a hydrothermal process, synthesize a SWAC/ZrO_2_–ZnO nanocomposite that efficiently breaks down 100 ppm of chlorpyrifos under UV light in 50 minutes at pH 6. A crystal size of 39.41 nm was confirmed by characterization techniques, and LC-MS analysis revealed that chlorpyrifos was fragmenting into smaller pieces. DFT simulations indicated the formation of reactive hydrogen bonds, and the degradation proceeded according to pseudo-first-order kinetics. According to^166^ study the NiO–ZnO nanocomposite's photocatalytic performance for breaking down the herbicide bentazon under UV light after 100 minutes of exposure. The study found that bentazon had a 70% degradation efficiency. This suggests that the nanocomposite may be useful in breaking down pesticides in water.^167^ Investigated the photocatalytic degradation of the pesticide lambda-cyhalothrin (LCY) using cerium-doped ZnO nanocomposites in the presence of natural sunlight. The coprecipitation method was used to synthesize Ce–ZnO, and methods like PXRD, SEM, FTIR, and EDAX were used to characterize its properties. The nanocomposites demonstrated high photocatalytic efficiency, degrading approximately 92% of LCY under ideal conditions (100 ppm initial concentration, 20 mg catalyst dose, and a UV index of 10–11) after three hours of exposure to sunlight. The average crystallite size was 31.42 nm. A pseudo-first-order kinetic model described the degradation.

The efficiency of photocatalytic degradation, as outlined in Table 8, is influenced by several factors, including the type of catalyst, the structure of the pesticide, the source of irradiation, and the conditions of the reaction. Modified ZnO systems, which may be doped, defect engineered, or formed as composites, demonstrate enhanced activity due to better charge separation and an optimized band structure. When parameters such as catalyst dosage, pH, and exposure time are carefully controlled, these well-designed ZnO catalysts can achieve rapid pesticide removal, typically exceeding 80–95% efficiency under UV or visible light. Ultimately, the interplay between the light source and the design of the catalyst plays a crucial role in determining the degradation performance across various pesticide classes.

**Table 8:** Degradation of different pesticides using ZnO nano-photocatalyst

Photocatalyst	Pesticide	Efficiency (%)	Light source	Reaction condition	Ref.
ZnO/αFe_2_O_3_	Carbamate	89	Solar light	Pesticide dose = 5 g L^−1^, catalyst dose = 1 g L^−1^, time = 3 h, pH = 8.5	18
La-doped ZnO	2-Chlorophenol	83.92	Visible light	Pesticide dose = 10 ppm, catalyst dose = 10 mg, time = 2 h	131
Ag-doped ZnO	Carbaryl	98	UV light	Pesticide dose = 5 ppm, photocatalyst = 5 mg L^−1^, time = 60 min	132
ZnO NPs	*p*-Nitrophenol	92	UV light	Pesticide dose = 20 mg L^−1^, catalyst dose = 1.5 g L^−1^, time = 180 min	118
ZnO	Lambda-cyhalothrin	87	Solar light	Pesticide dose = 20 mL of 100 ppm, ZnO = 50 ppm, temperature = 31 °C, time = 30 min (dark), 60 min (light)	168
Fe–ZnO	Chlorpyrifos	67	Solar light	Pesticide dose = 5 mg L^−1^, pH = 8, time = 140 min	169
Cu–ZnO heterostructure	Chlorpyrifos	95	Solar light	Pesticide dose = 200 mg L^−1^, catalyst dose = 3 g L^−1^, pH = 6.0, time = 240 min	170
Ce–ZnO nanocomposites	Lambda-cyhalothrin	92	Solar light	Pesticide dose = 100 ppm, catalyst dose = 20 mg, time = 3 h	167
Pbi–ZnO–g-C_3_N_4_	Atrazine	85.3	Visible light	Catalyst dose = 216.40 g L^−1^, time = 260 min	171
ZnO/rGO	Metalaxyl	90.25	UV light	Pesticide dose = 10 mg L^−1^, catalyst dose = 0.75 g L^−1^, pH = 7, time = 120 min, UV intensity = 220 MW cm^−2^	172
ZnO/rGO	Metalaxyl (real agricultural runoff)	51.17	UV light	Pesticide dose = 10 mg L^−1^, catalyst dose = 0.75 g L^−1^, pH = 7, time = 120 min, UV intensity = 220 MW cm^−2^	
rGO/Fe_3_O_4_/ZnO	Metalaxyl	92.11	Visible light	Time = 120 min, order of reaction = 1^st^ order kinetic model	173
ZnO·WO_3_ composite	Paraquat dichloride	88.3	UV light	Pesticide dose = 35 mg L^−1^, catalyst dose = 0.04 g, pH = 9, temperature = 40 °C, cycles of reaction = 3	174
ZnO	Methamidophos	86.66	UV light	Pesticide dose = 50 mg L^−1^, catalyst dose = 3 g L^−1^ with ultra-pure water	175
ZnO	Methamidophos	57.95	UV light	Pesticide dose = 50 mg L^−1^, catalyst dose = 3 g L^−1^ with river water	
Fe_2_O_3_–ZnO	Profenofos	100	Dark	Pesticide dose = 1825 mg L^−1^, time = 60 min	176
CuO–ZnO nanocomposite	Profenofos	100	UV light	Pesticide dose = 1215 mg L^−1^, time = 80 min	177
ZnO/Cu/GO	Quinalphos	99	Visible light	Pesticide dose = 40 ppm, catalyst dose = 3 mg L^−1^, time = 20 min, pH = neutral	178
PANI/ZnO–CoMoO_4_	Imidacloprid	97	Visible light	Pesticide dose = 4.5 ppm, catalyst dose = 163.5 mg, time = 180 min, pH = 4	179
ZnO/rGO	Dimethoate	99	UV light	Pesticide dose = 5 mg L^−1^, catalyst dose = 50 mg, light intensity = 2.45 mW cm^−2^, time = 180 min	180
ZnO/CoFe_2_O_4_	Imidacloprid	98.1	Visible light	Pesticide dose = 5 ppm, catalyst dose = 0.05 g, pH = 10	181

## 9. Conclusion

This document reveals the distinctive electrical and structural properties of ZnO-based photocatalysts, which include a direct band gap, favorable band-edge positions, and defect-induced donor states. These characteristics contribute to their exceptional ability to degrade various pesticide contaminants. Although other photocatalytic materials offer certain benefits, ZnO surpasses them in oxidative strength, charge mobility, efficiency of electron–hole separation, and surface reactivity, especially when exposed to UV light. Additionally, recent advancements in ZnO engineering such as metal and non-metal doping, defect manipulation, heterojunction formation, plasmonic coupling, and photothermal integration have substantially enhanced its light-harvesting efficiency, charge transfer dynamics, and ROS generation. These enhancements effectively mitigate ZnO's inherent challenges, including photocorrosion and limited sensitivity to visible light, thereby improving its applicability in real-world wastewater treatment scenarios.

This document specifically pinpoints the potential environmental benefits of ZnO based nanomaterials in agriculture, particularly through photocatalytic processes that can eliminate pesticide-laden runoff, thereby minimizing groundwater contamination and protecting irrigation sources and aquatic ecosystems. This not only supports safe food production but also alleviates ecological pressures on soil microorganisms, aquatic flora, and beneficial insect populations. As the global community strives for low-impact and energy-efficient water treatment solutions, ZnO-based photocatalytic systems emerge as a promising avenue for developing sustainable, climate-resilient, and environmentally responsible remediation technologies.

Despite these promising results, current research on ZnO nanomaterials for wastewater treatment, particularly in pesticide degradation, reveals several critical areas or limitations for further exploration to improve their effectiveness and practical application. One key challenge is to enhance the long-term stability and reusability of ZnO photocatalysts, which are crucial for achieving sustainable and cost-effective solutions. While ZnO has shown potential in laboratory settings, there is a significant gap in studies that combine ZnO photocatalysis with other wastewater treatment techniques, such as biological treatments or membrane filtration, creating hybrid systems that integrate these methods could optimize pollutant removal and broaden the application of ZnO in real-world scenarios. Additionally, research into combining ZnO with 2D materials like black phosphorus and carbon nitride could enhance photocatalytic efficiency by improving light absorption and promoting effective charge separation, addressing some of ZnO's inherent limitations.

Future research should concentrate on integrating these strategies by advancing the design of ZnO nanostructures through controlled defects, facet engineering, and lattice strain, which can improve charge separation and broaden activity into the visible light spectrum. Additionally, combining ZnO with plasmonic metals, carbon materials, or narrow band-gap semiconductors can enhance solar absorption and increase degradation efficiency.

The photocorrosion of ZnO in acidic environments necessitates ongoing efforts to enhance its stability, with effective strategies including surface passivation, core–shell structures, and protective coatings. Additionally, green synthesis methods utilizing plant extracts or biopolymers promote environmentally friendly and scalable production processes. The use of immobilized ZnO in forms such as membranes, coatings, and 3D-printed structures is expected to become increasingly prevalent, enabling catalyst recovery and sustained operation in practical applications. Furthermore, integrating ZnO with photothermal materials, adsorption components, solar concentrators, or LED-powered systems presents promising opportunities for the remediation of agricultural runoff in real-world settings.

## Conflicts of interest

Authors declare no conflict of interest.

## Data Availability

No primary research results, software or code have been included and no new data were generated or analysed as part of this review.
